# Characterization of silk sutures coated with propolis and biogenic silver nanoparticles (AgNPs); an eco-friendly solution with wound healing potential against surgical site infections (SSIs)

**DOI:** 10.3906/sag-1906-48

**Published:** 2020-02-13

**Authors:** Tuba BAYGAR

**Affiliations:** 1 Research Laboratories Center, Muğla Sıtkı Koçman University, Muğla Turkey

**Keywords:** Propolis, silver nanoparticle, suture, antibacterial, wound healing, cytotoxicity

## Abstract

**Background/aim:**

Bacterial adherence to a suture material is one of the main causes of surgical site infections. An antibacterial suture material with enhanced wound healing function may protect the surgical site from infections. Thus, the present study aimed to investigate the synergistic effect of propolis and biogenic metallic nanoparticles when combined with silk sutures for biomedical use.

**Materials and methods:**

Silver nanoparticle (AgNP) synthesis was carried out via a microbial-mediated biological route and impregnated on propolis-loaded silk sutures using an in situ process. Silk sutures fabricated with propolis and biosynthesized AgNPs (bioAgNP-propolis-coated sutures) were intensively characterized using scanning electron microscopy (SEM), energy dispersive X-ray spectroscopy (EDS), thermogravimetric analysis (TGA), and differential scanning calorimetry (DSC). The antibacterial characteristics of the bioAgNP-propolis-coated sutures were evaluated using the agar plate method. The biocompatibility of the bioAgNP-propolis-coated sutures was evaluated using 3T3 fibroblast cells, and their wound-healing potential was also investigated.

**Results:**

BioAgNP-propolis-coated sutures displayed potent antibacterial activity against pathogenic gram-negative and gram-positive bacteria, *Escherichia coli* and *Staphylococcus aureus*, respectively. BioAgNP-propolis-coated silk sutures were found to be biocompatible with 3T3 fibroblast cell culture. In vitro wound healing scratch assay also demonstrated that the extract of bioAgNP-propolis-coated sutures stimulated the 3T3 fibroblasts’ cell proliferation.

**Conclusion:**

Coating the silk sutures with propolis and biogenic AgNPs gave an effective antibacterial capacity to surgical sutures besides providing biocompatibility and wound healing activity.

## 1. Introduction

Surgical site infections (SSIs) are common complications that occur after surgery. The surgical suture can itself be the main cause of the SSI owing to microbial adherence [1]. Microbial accumulation onto the suture is related not only to the microbial species but also the structure and chemical composition of the suture material [1–3]. Biofilm formation occurs after the suture material becomes contaminated, and it cannot be eradicated by biologic agents, chemical agents, or other mechanisms of wound decontamination [2]. 

Multifilament sutures, e.g., silk, are preferred to monofilament sutures because of the easy manipulation and knot security. However, some studies reported that multifilament sutures are known to lead to bacterial adherence, which can cause severe inflammations [4].

Recently, a new generation of suture materials with improved physical and biological activities have been developed. Modification of the suture materials with antimicrobial agents is a popular research area that has been accepted as an important approach for the prevention of wound infections [5].

Multidrug-resistant bacteria, most of which are associated with nosocomial infections, are a current major risk to public health [6]. There is a growing interest in invention of new biocidal agents to avoid the antimicrobial resistance.

Propolis is a natural product that has been widely used as a folk medicine all around the world. Antiinflammatory and wound healing potentials and antimicrobial activity of propolis against different microbial strains have been reported [7–10]. 

Over the past decade, silver nanoparticles (AgNPs) have been the most intensively investigated metallic nanoparticles, and their broad-spectrum antimicrobial potentials have been reported by various authors [11–13]. 

Green nanosynthesis methods are known to be nontoxic, environmentally safe, and cost- and time-saving procedures that provide appreciable results for nanotechnology [14]. Novel green synthesis routes that are known to be encouraging approaches for reducing metals by specific metabolic pathways utilize biological organisms, e.g., microorganisms [15]. 

Synergistic antibacterial effect of propolis and AgNPs with an enhanced wound healing capacity might be a novel approach to develop new suture materials. For this purpose, nonabsorbable silk sutures were initially treated with propolis using a slurry dipping technique. Following the propolis coating, silver nanoparticles that were bioynthesized using the cell-free extract of a bacteria, *Streptomyces* sp. AU2, have been deposited onto the propolis-loaded sutures via an in situ process. Morphology and elemental composition of bioAgNP-propolis-coated sutures were evaluated by scanning electron microscopy (SEM) and energy dispersive X-ray spectroscopy (EDS). Thermal characterization was also performed using thermogravimetric analysis (TGA) and differential scanning calorimetry (DSC). The antibacterial feature of the bioAgNP-propolis-coated silk sutures was determined against pathogenic bacteria. The cytotoxicity and in vitro wound healing capacity of the bioAgNP-propolis-coated silk sutures have also been evaluated using NIH 3T3 murine fibroblast cell culture.

## 2. Materials and methods

### 2.1. Preparation of the bioAgNP-propolis-coated sutures

#### 2.1.1. Propolis-loading of the sutures

Propolis used within the present study was obtained from Köyceğiz, Muğla, Turkey and extracted with methanol. Nonabsorbable 4.0 silk sutures obtained from a suture-producing company (Doğsan, İstanbul, Turkey) were used as the base material for designing the bioAgNP-propolis coating. Silk sutures were dipped in propolis solutions for 2 min, and dried for 24 h [16]. The weight of propolis coatings on sutures was measured by an electronic balance (M Power, Sartoius Instruments Ltd., UK), and the propolis concentration per unit of length (centimeter) was calculated. 

#### 2.1.2. AgNP immobilization to propolis-loaded sutures

Biosynthesis of silver nanoparticles was performed according to the green synthesis method that was previously published [17]. For bioAgNP immobilization onto the propolis-loaded sutures, propolis-loaded suture fragments of 10 pieces (1 cm), cell-free supernatant (10 mL), and AgNO3 solution (1 mM) (50 mL) were incubated at 28 °C in an orbital shaker (130 rpm) for 24–48 h [18]. 

### 2.2. Characterization of the bioAgNP-propolis-coated sutures

#### 2.2.1. Morphological and microanalytical characterization

Surface morphology of the bioAgNP-propolis-coated sutures was evaluated using SEM (JSM 7600F, JEOL, Japan), and elemental composition was determined by EDS (Oxford Instruments, UK) combined with SEM.

#### 2.2.2. Thermogravimetric analysis (TGA)

TGA of the bioAgNP-propolis-coated sutures was performed on a TGA instrument (Perkin Elmer TGA 4000, Perkin Elmer, Waltham, MA, USA). Samples were heated from 30 °C to 800 °C at a rate of 20 °C min−1 under a nitrogen flow rate of 20 mL min−1. Noncoated silk sutures were used as a control.

#### 2.2.3. Differential scanning calorimetry (DSC)

DSC of bioAgNP-propolis-coated sutures was carried out using a DSC instrument (Perkin Elmer DSC 8000, Perkin Elmer, Waltham, MA, USA). Suture fragments were placed in sealed aluminum pans and heated at 10 °C min−1 under a nitrogen atmosphere (flow rate 30 °C min−1) in the 20–550 °C range. Noncoated silk sutures were used as a control.

### 2.3. Antibacterial activity

Antibacterial activity of the bioAgNP-propolis-coated sutures was determined against gram-negative and gram-positive bacteria, *Escherichia coli* ATCC 25922 and *Staphylococcus aureus* ATCC 25923, using the standard agar plate method [19]. Experiments were performed in triplicate and the mean values ± standard deviation (SD) of the tests was calculated.

### 2.4. Biocompatibility/cytotoxicty

To evaluate the biocompatibility of the bioAgNP-propolis-coated sutures, NIH 3T3 murine fibroblast cell line obtained from ATCC (American Type Cell Culture) (Manassas, VA, USA) was used. 

#### 2.4.1. Extraction method

Depending on the standard protocols reported by ISO 10993-5, an indirect extraction method was performed to assess the in vitro cytotoxicity of bioAgNP-propolis-coated sutures [18]. 3T3 fibroblasts were incubated with the suture extract medium throughout time intervals of 1, 4, 8, and 10 days at 37 °C. The cell viability was evaluated using MTT assay [20] at 570 nm using a microplate reader (Thermo Scientific Multiskan FC, Thermo Fischer, Vantaa, Finland). Fresh culture medium without test materials was used as a negative control. Cell inhibition rate (%) was determined using the formula:

(% Cell Inhibition) = [100 × (Sampleabs)/ (Controlabs)] (1)

### 2.5. In vitro scratch wound healing assay

A scratch wound assay which measures the expansion of a cell population on surfaces was used to assess the spreading and migration capabilities of 3T3 fibroblasts. An in vitro scratch assay was used to evaluate the wound healing effect of the bioAgNP-propolis-coated sutures [21]. Cells were treated with the extract of the bioAgNP-propolis-coated sutures prepared as above, and the control group was prepared as the cells cultured in the basal medium. Representative images from each cell culture dish of the scratched areas were photographed using a Leica DM IL microscope (Leica Microsystems, Wetzlar, Germany) to estimate the relative migration of the cells. Experiments were performed in triplicate and the mean values ± standard deviation (SD) of the tests was calculated.

## 3. Results

**Figure 1:**
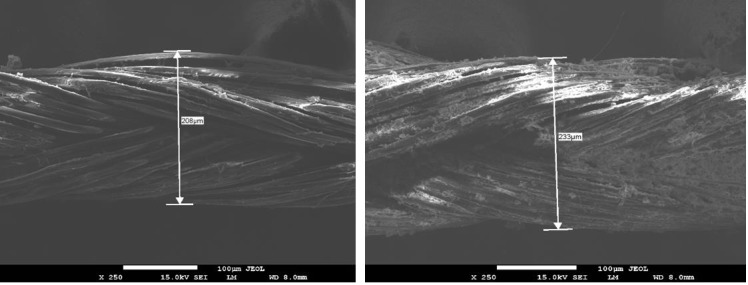
SEM micrographs of control suture (left) and bioAgNP-propolis-coated suture (right). The bar represents 100 μm.

**Figure 2:**
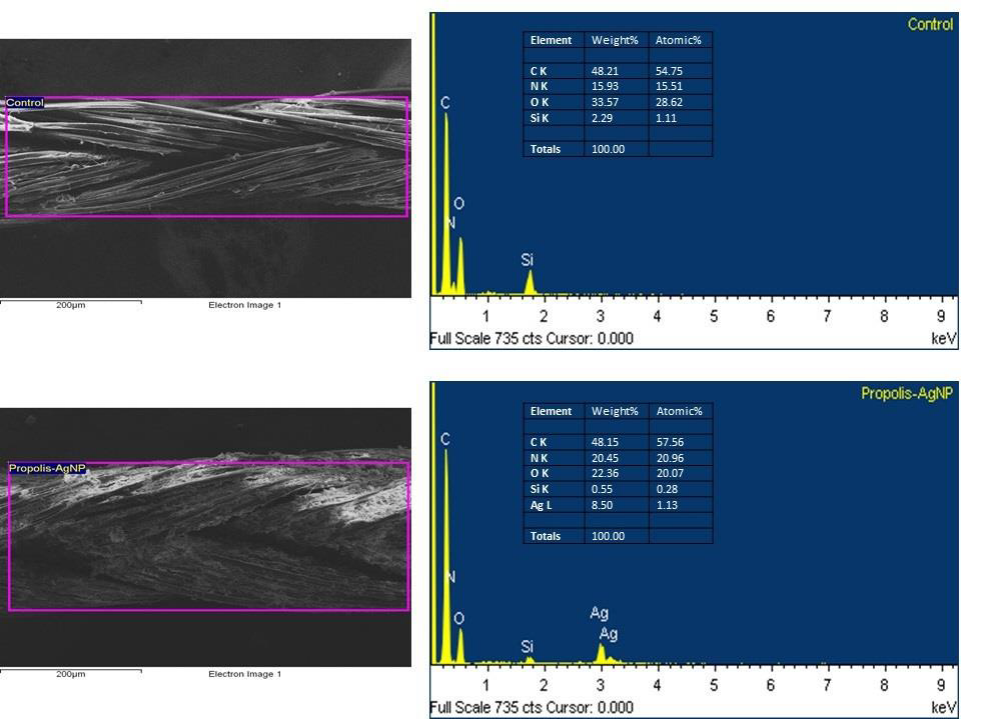
EDS spectrums of control suture and bioAgNP-propolis-coated suture.

Initial qualitative analysis of the morphology of bioAgNP-propolis-coated sutures was conducted by SEM. SEM micrographs of the samples displayed the typical multifilament structure of the silk sutures. Propolis-AgNP deposition on the surface of the bioAgNP-propolis-coated sutures was observed from the images. The coating process used a slurry dipping technique to generate propolis-loaded sutures of approximately 40 μg/cm. The transverse measurements of the sutures were found to be 208 µm and 233 µm for control and bioAgNP-propolis coated-sutures, respectively (Figures 1a and 1b). The presence of the silver ions was detected by EDS analysis for coated sutures (Figure 2a), and no silver peak was observed in the EDS spectrum of the control group (Figure 2b). 

**Figure 3:**
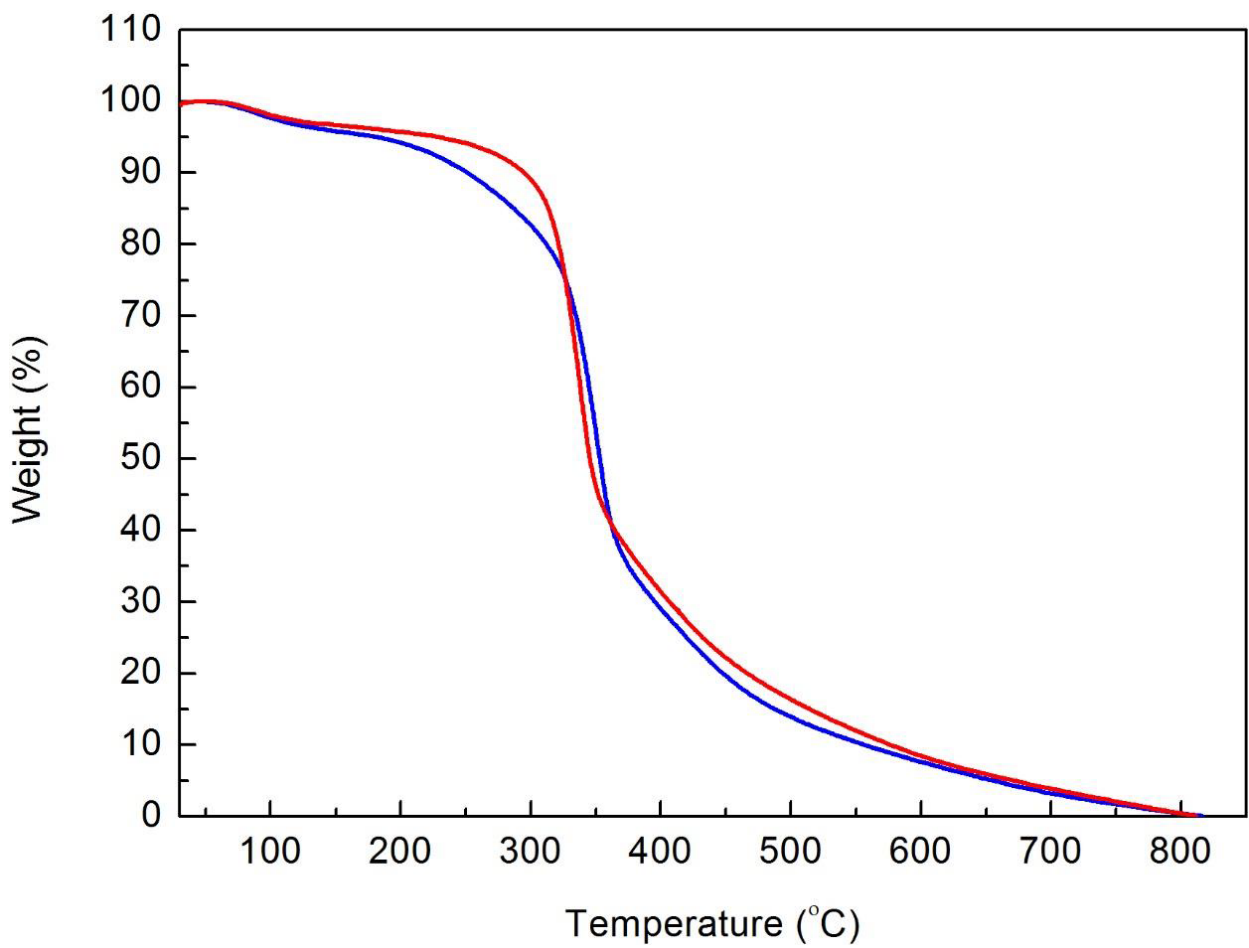
Thermogravimetic analysis of bioAgNP-propolis-coated sutures (blue line)and noncoated sutures (control group, red line).

The thermal decomposition graphics for the bioAgNP-propolis-coated sutures and noncoated silk sutures (control) are shown in Figure 3. The bioAgNP-propolis-coated sutures showed increased thermal stability, with a mass change of around 51.16% from 250 °C to 465 °C. 

**Figure 4:**
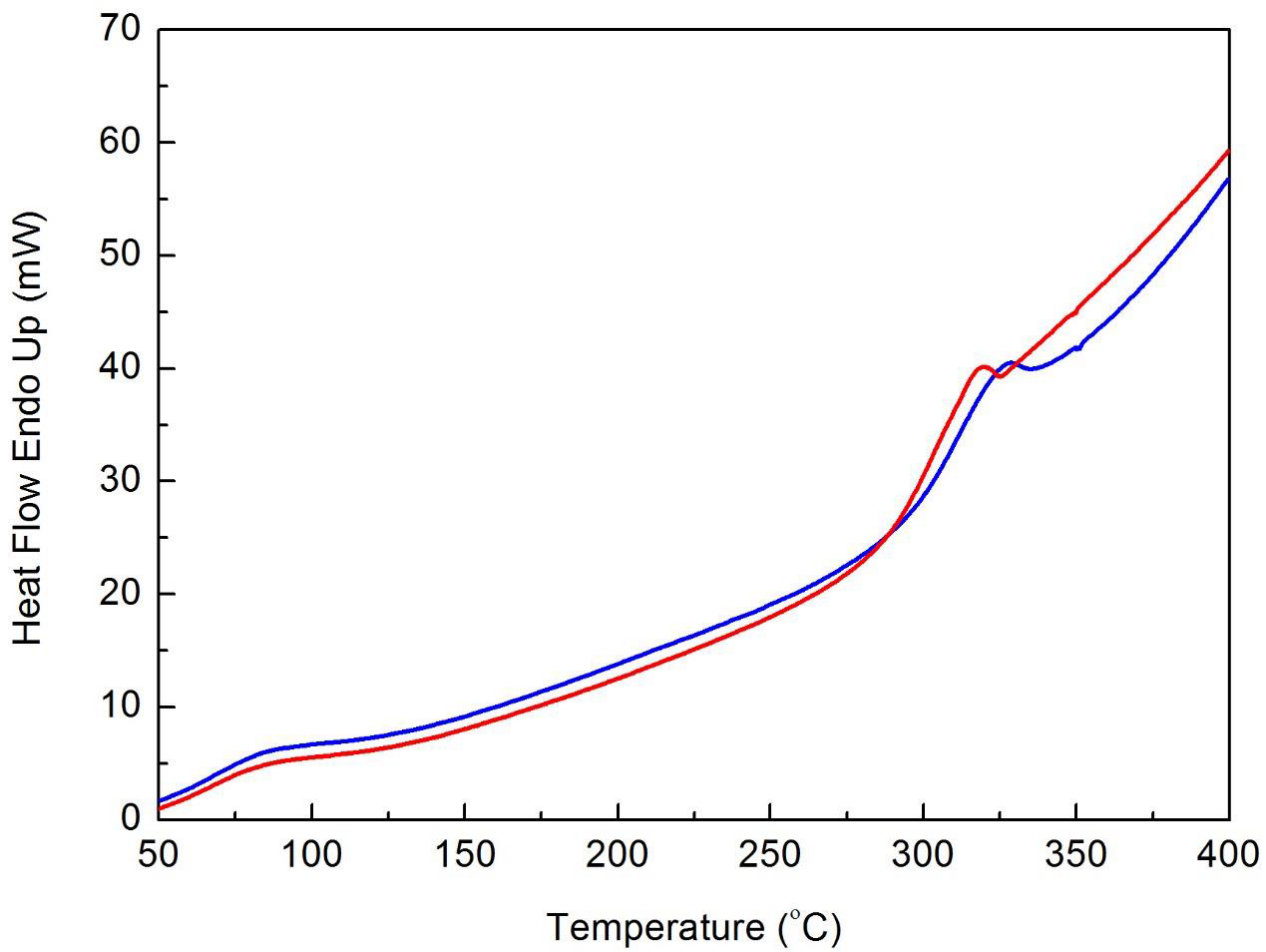
Differential scanning calorimetry curves of bioAgNP-propolis-coated sutures (blue line) and noncoated sutures (control group, red line).

According to the DSC analysis, shapes of hear flow curves appeared to be slightly affected by the bioAgNP-propolis coating process (Figure 4).

**Figure 5:**
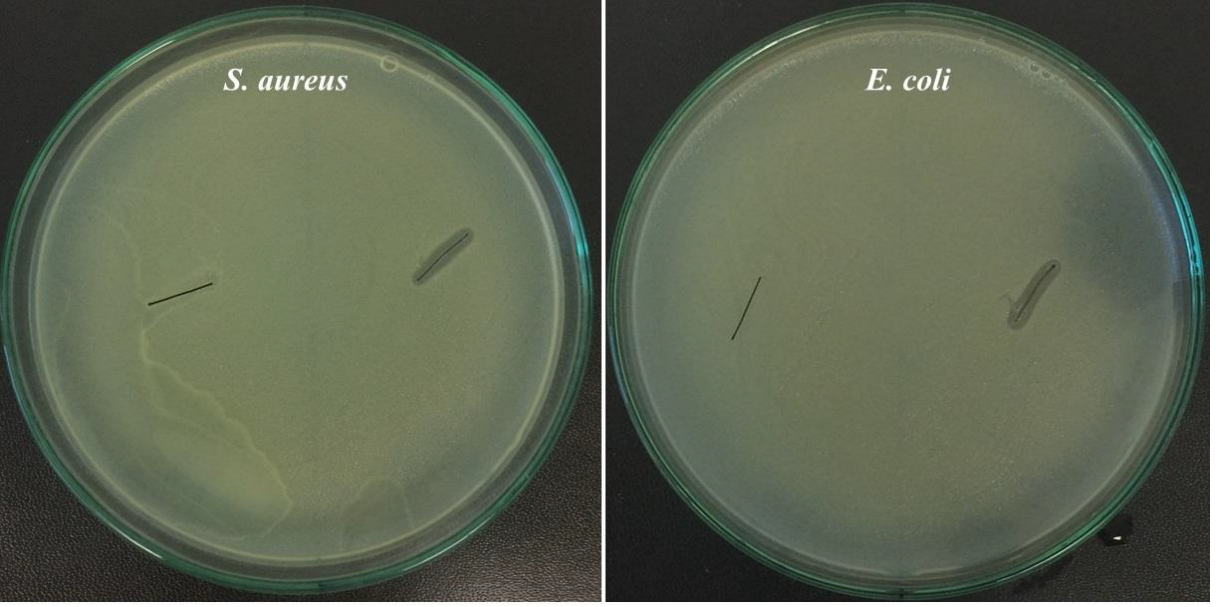
Antibacterial activity of bioAgNP-propolis-coated sutures against S. aureus and E. coli.

The antibacterial features of the bioAgNP-propolis-coated sutures were evaluated against pathogenic bacteria (Figure 5). 

**Figure 6:**
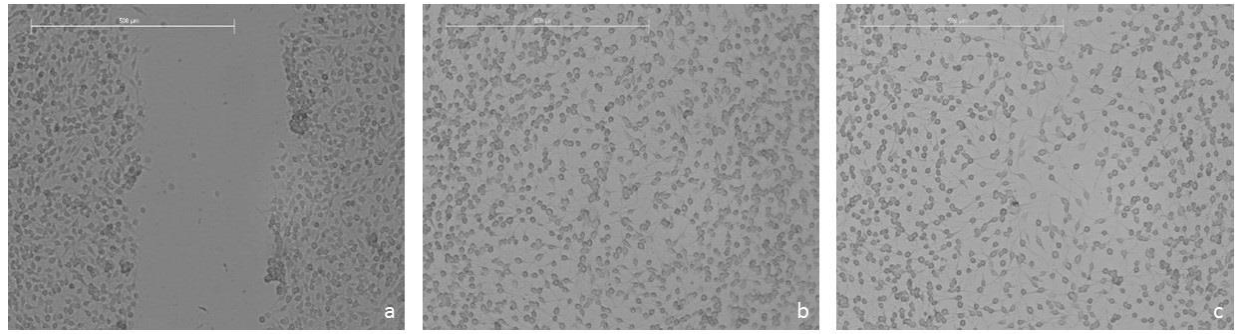
Images of scratch wound healing assay: a) 0 h, b) basal cell culture medium after 24 h, and c) extract of bioAgNPpropolis- coated suture after 24 h. The bar represents 500μm.

The wound-healing activity of the bioAgNP-propolis-coated sutures was evaluated using 3T3 fibroblasts. The proliferation and migration of the fibroblasts were similar to the control group, which was incubated with basal cell culture medium. The cells stimulated the cell migration after 24 h (Figure 6). 

**Figure 7:**
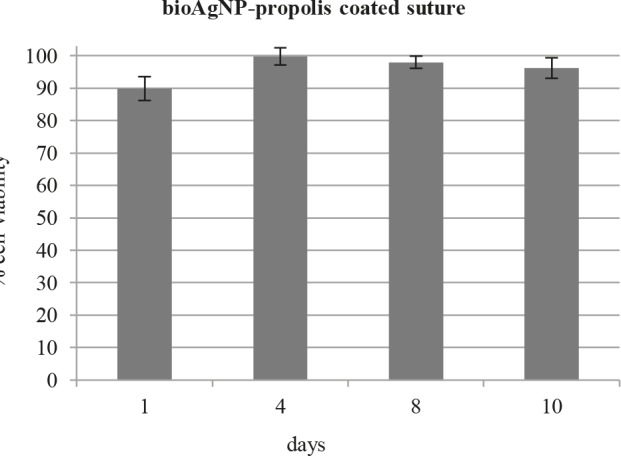
Cell viability of 3T3 fibroblasts treated with propolisbioAgNP- coated suture extracts at 1, 4, 8, and 10 days (reported as a percentage of the negative controls).

Biocompatibility analysis applied using an indirect method indicated that bioAgNP-propolis-coated sutures did not display significant adverse effect on cell viability of the 3T3 fibroblasts (Figure 7). 

## 4. Discussion

In the present study, antibacterial silk sutures enhanced with propolis and biogenic silver nanoparticles were prepared and characterized. To date, no other report has been found to generate silk sutures with propolis and silver nanoparticles.

For microanalytical characterization of new generated sutures, the impregnation of silver nanoparticles was obvious when on the SEM-EDS spectrum. Similar EDS spectrum results were also obtained by Dhas et al., who coated the silk fibers with silver nanoparticles using an aqueous extract of a plant, *Rhizophora apiculata *leaf [22].

According to the TGA graphics of both the noncoated and coated suture groups, the initial weight loss below 100 °C is supposed to be caused by the evaporation of water [23]. The decomposition stage of the control group was marked at 250 °C–465 °C, with a total mass change of 56.99% observed. Elakkiya et al. indicated that the weight loss was due to thermal decomposition of the antiparallel β-sheet structure of fibroin, which forms the structural core of silk [24]. The increased thermal stability of the bioAgNP-propolis-coated sutures could be due to the propolis extract, and also the bioorganic components present in the cell-free extract of *S.* *griseorubens *AU2 strain. Similar results were also reported by Dhas et al., who fabricated silk fibers with AgNPs synthesized via a plant extract [22]. 

DSC is a technique used for the thermal characterization of materials and helps to establish a connection between the specific physical properties of substances and the temperature [25]. In a report by Ho et al., DSC curves of the raw silk fiber displayed 2 broad endothermic peaks at around 100 °C and 365 °C, which were attributed to the loss of moisture, and thermal degradation of a well-oriented β-sheet crystalline conformation, respectively [23]. They also reported that the endotherm and exotherm peaks at about 225 °C and 270 °C might be attributed to the molecular motion within the α-helix crystals and to the crystallization during heating by forming the β-sheet structure from a random-coil conformation, respectively.

When compared with the noncoated silk sutures, the antibacterial activity of bioAgNP coated sutures was found to be enhanced by synergistic effect propolis and silver nanoparticles. Concerning the Standard SNV 195920–1992, a material is considered to have good antibacterial potential when the zone of inhibition measurement is higher than 1 mm [22,26]. The clear growth inhibition area around the bioAgNP-propolis-coated sutures indicated an effective antibacterial capability. *E. coli *and *S. aureus* are known to be multiresistant bacteria that are responsible for nosocomial infections [27]. Propolis is well-known for its antimicrobial potential [28,29]. Bacteriostatic activity of propolis components, polyphenols, and flavonoids was also indicated by Tosi et al. against *E. coli* and *S. aureus *[30]. Within the present study, it was clearly revealed that coating sutures with propolis and biosynthesized AgNPs can be suggested as a preventive method to protect the surgical site from bacterial biofilm formation which might be due to the synergistic effect of propolis and AgNPs. There are other reports that figured out the antimicrobial action of the AgNP coated sutures. In a study by Pratten et al., who coated silver-doped bioactive glass (AgBG) onto the silk sutures, it was reported that AgBG coating limited the *S. epidermidis* attachment [31]. Similarly, Dhas et al. figured out that Ag-coated silk fibers exhibited more than 90% inhibition against *Pseudomonas aeruginosa *and *S. aureus *[22]. There are some other reports that studied the antimicrobial activity of Ag coated sutures. De Simone et al. coated the silk sutures with AgNPs obtained by photoreduction of a silver solution using an ultraviolet (UV) lamp, and the antibacterial activity analysis of the coated sutures similarly indicated the good efficacy of the sutures against *E. coli* and *S. aureus *[32]. It is reported that an interaction occurs between the AgNPs and bacterial membrane, and AgNPs penetrate the cell, which causes a potent disturbance regarding normal cell function, structural damage, and cell death [33]. Different responses given to the toxicity of AgNPs that are displayed by different bacterial species are related to the composition of the bacterial cell wall [22].

Similar to any complex pathophysiological mechanism, the wound-healing process includes coagulation, inflammation, cellular proliferation, and matrix and tissue remodeling stages [34]. Opportunistic pathogenic microorganisms that cause wound infections have recently become an important issue through the healthcare practices [35,36]. Incorporation of AgNPs within biomaterials resulted in promising results for enhanced wound healing management [37]. The positive effects of silver nanoparticles through their antimicrobial properties, reduction in wound inflammation, and modulation of fibrogenic cytokines were also reported by Tian et al. [38]. Gallo et al., who performed in vitro scratch assay using the degradation eluates of silver-treated PLGA sutures, found that the presence of silver promoted cell migration and proliferation of 3T3 murine fibroblasts in the wound area [39].

The cytotoxicity study of bioAgNP-propolis coated sutures was evaluated by MTT cell metabolism assay using 3T3 fibroblasts. Similar to the obtained results, Dhas et al. demonstrated that 3T3 fibroblasts displayed ∼90% viability after 5 days of incubation with the AgNP-impregnated silk fibers [22]. The biocompatibility evaluation of absorbable PLGA sutures functionalized with silk sericin and silver also indicated that 3T3 fibroblasts cell viability and proliferation were not significantly affected by silver presence [40].

Practical applications of modified sutures are gaining much importance for the treatment of wounds that have potential infection risk. In the present study, propolis-treated silk sutures were coated with biogenic silver nanoparticles through a deposition technique. Characterization studies revealed the effective silver deposition onto the surface of the sutures. The antibacterial capability that was demonstrated on pathogenic bacteria confirmed the strong capability of the propolis-silver treatment. A cytotoxicity test performed on the extract of bioAgNP-propolis-coated sutures demonstrated that bioAgNP-propolis-coated sutures might be used without adverse effects on normal cells. The results obtained by the scratch assay treated with the bioAgNP-propolis-coated suture extract revealed that its presence promoted cell migration and proliferation in the wound area. The present research indicated that naturopathic antibacterial coatings developed on silk sutures may offer beneficial advantages in terms of prevention from surgical infections and enhancing the wound healing process. With the enhanced synergistic effect of a natural drug and a metallic nanoparticle, a coating process with propolis and biogenic AgNPs can be suggested as a novel approach towards antibacterial biomaterials for biomedical use and clinical practice.

## Acknowledgments

The author is thankful to Prof. Dr. Aysel Uğur and Assoc. Prof. Dr. Nurdan Saraç for their kind assistance.
